# Selective Loss of Responsiveness to Exogenous but Not Endogenous Cyclic-Dinucleotides in Mice Expressing STING-R231H

**DOI:** 10.3389/fimmu.2020.00238

**Published:** 2020-02-21

**Authors:** Melissa M. Walker, Soojin Kim, William J. Crisler, Kimberlie Nguyen, Laurel L. Lenz, John C. Cambier, Andrew Getahun

**Affiliations:** ^1^Department of Immunology and Microbiology, University of Colorado SOM, Aurora, CO, United States; ^2^Department of Biomedical Sciences, National Jewish Health, Denver, CO, United States

**Keywords:** STING, cGAS, cyclic dinucleotide, allelic variants, infection, cancer, immunotherapy

## Abstract

Stimulator of interferon genes (STING) plays a central role in innate immune responses to viral and intracellular bacterial infections, and cellular damage. STING is a cytosolic sensor of cyclic dinucleotides (CDNs) including those produced by pathogenic bacteria and those arising endogenously as products of the DNA sensor cGAS (e.g., 2′3′ cGAMP). The two most common alternative allelic variants of STING in humans are STING-R71H-G230A-R293Q (STING-HAQ) and STING-R232H that are found in 20.4% and 13.7–17.6% of the population, respectively. To determine the biologic consequences of these genotypic variations, we generated knock-in mice containing the murine equivalents of each variant and studied their responsiveness to CDNs. Homozygous STING-HAQ (R71H-I229A-R292Q) and STING-R231H mice were found to be unresponsive to all exogenous CDNs tested (ci-di-GMP, ci-di-AMP, 3′3′ cGAMP and Rp,Rp-CDA). Responses of homozygous STING-HAQ mice to endogenous 2′3′ cGAMP was also greatly impaired. However, homozygous STING-R231H mice are fully responsive to 2′3′ cGAMP. Analysis of heterozygous mice revealed reduced responsiveness to exogenous and endogenous CDNs in mice carrying a single copy of STING-HAQ, while STING-R231H heterozygous mice exhibit reduced responsiveness to exogenous but not endogenous CDNs. These findings confirm and extend previous reports by demonstrating differing impact of allelic variation of STING on the ability to sense and respond to exogenous vs. endogenous CDNs. Finally, the STING-R231H variant mouse represents a useful tool with which to examine the relative contributions of STING sensing of exogenous and endogenous CDNs in the context of bacterial infections and CDN-based cancer immunotherapeutics.

## Introduction

Stimulator of interferon genes (STING, aka TMEM173, MPYS, MITA) (1–3) is a cytosolic receptor for bacterial and synthetic cyclic dinucleotides (CDNs) (4–6), and the endogenous mammalian CDN 2′3′ cGAMP (7, 8) generated by cGAS upon sensing of cytoplasmic DNA. Ligand binding activates several pathways, including those involving IRF3 and NF κB, resulting in production of proinflammatory cytokines (2, 9). STING activation can also lead to autophagy (10–12).

STING plays an important role in the immune response to viral and intracellular bacterial pathogens (13, 14), autoimmune inflammation (15, 16), and immune surveillance (17, 18) [see (19, 20) for a comprehensive review of STING biology]. Bacterial CDNs and synthetic derivatives comprise a potent new class of adjuvants that evoke protective humoral and CD4 and CD8 T cells responses in a STING-dependent manner (21–24). In particular, the use of synthetic CDNs in combinational anti-cancer immunotherapy has provided encouraging results (6, 25).

Several allelic variants of STING can be found in the general population (26), some of which appear to have altered function. The two most common minor allelic variants are R232H and R71H-G230A-R293Q (HAQ). The prevalence of these alleles varies among ethnic populations, but on average 20.4% of the human population carries at least one copy of the HAQ variant. The prevalence of R232H variant is reported to be 13.7–17.6% (27–29). To gain insight into the functional effects of these variations, several groups have used ectopic overexpression systems, sometimes reaching conflicting conclusions. Human STING HAQ has been reported to have decreased to absent responsiveness to exogenous CDNs (27), while both normal (27) and absent responses (28) to 2′3′ cGAMP have been reported. Human STING-R232H was reported to have reduced responsiveness to most exogenous CDNs but relatively normal responsiveness to 2′3′ cGAMP (27, 28), although others have reported reduced responsiveness to 2′3′ cGAMP (30). Selective unresponsiveness to exogenous but not endogenous CDNs was also observed in mutational analysis of murine STING in which a R231A substitution (equivalent position to R232 in humans) caused a loss of responsiveness bacterial CDNs but not 2′3′ cGAMP (4, 7). A key difference between endogenous 2′3′ cGAMP and bacterial derived CDNs is that while the latter only uses 3′-5′ phosphodiester bonds to link nucleotides, the former uses a 2′-5′ phosphodiester bond and a 3′-5′ phosphodiester bond (7, 30). This altered structure allows 2′3′ cGAMP to interact with STING with a higher affinity, which may explain differences in binding to allelic variants (30, 31).

To gain more insight into the effects of these two allelic variants on STING function under physiological conditions we generated two new mouse models in which we introduced point mutations in mouse STING in order to emulate the two most common STING variants: HAQ and R232H. *Ex vivo* and *in vivo* analysis of immune responses to exogenous and endogenous CDNs reveal that neither variant responds to the exogenous CDNs tested. Interestingly, while STING HAQ has a largely impaired ability to sense endogenous CDNs, STING R231H responds normally to the cGAS product 2′3′ cGAMP.

## Materials and Methods

### Mice

Eight- to sixteen-week-old mice were used for all experiments. STING KO mice (32, 33) and STING HAQ knock-in mice (29) were generated as previously described. C57BL/6 mice were purchased from the Jackson Laboratory and used as WT controls. The STING R231H knock-in mice were generated by the National Jewish Health Mouse Genetics Core Facility as described below. The STING-HAQ mice and STING-R231H mice are available at The Jackson Laboratory as Stock No. 034434 and 034435, respectively. Both male and female mice were used in our *in vitro* and *in vivo* experiments. We have not observed any difference in the response to CDNs between the sexes. Mice were housed and bred in the Animal Research Facilities at the University of Colorado Anschutz Medical Campus and National Jewish Health. All experiments were performed in accordance with the regulations and approval of University of Colorado and National Jewish Health Institutional Animal Care and Use Committee.

### Generation of the STING R231H Knock-In Mice

A similar approach as described for the HAQ knock-in mice (29) was used to generate the R231H knock-in mice. A linearized targeting vector (Figure S1A) which covers ~10 kb of the genomic region in the *STING* locus on mouse chromosome 18, with the R231H point mutation, was transfected into C57BL/6-derived JM8.A3 embryonic stem cells. Clones were selected for neomycin positive and diphtheria toxin negative clones. Successful targeting was assessed by PCR. Positive clones were injected into C57BL/6J blastocytes and implanted. Chimerism was determined by coat color and males with high chimerism were bred with female C57BL/6 females to select for germline transmission. Successful germline transmission was confirmed by DNA sequencing. These mice were crossed with the FLP1 recombinase line (B6;SJL-Tg(ACTFLPe)9205Dym/J, Jackson Laboratory, ME.) to remove the neo gene, generating the STING R231H knock-in line. To validate successful introduction of the R231H generating point mutation exon 6 was amplified by PCR (Exon 6-F, 5′-TCACACTGAGAAGGCTAACGAGC-3′; Exon 6-R, 5′-CACCATAGAACAGGGATCACGC-3′) and sequenced (Figure S1B).

### *In vitro* Stimulation With CDN

Single cell suspensions of splenic cells were prepared. In some experiments red blood cells were lysed using ammonium chloride TRIS. In most experiments, live cells were isolated using Lympholyte M kit (Cedarlane, Burlington, Ontario, Canada) and B cells were purified by CD43 negative selection using MACS CD43-microbeads (Miltenyi Biotec Inc., San Diego, CA.). Resultant populations were routinely >97% B cells based on B220 staining and FACS analysis. *Ex vivo* B cells and splenocytes were cultured in IMDM supplemented with 10% FBS, sodium pyruvate (1 mM), L-glutamine (2 mM), 1% penicillin/ streptomycin, 2-ME (50 μM), HEPES buffer (10 mM), and 1% non-essential amino acids.

*Ex vivo* B cells or splenocytes were seeded (3 × 10^5^ cells/100 μL/well) in 96 well flat-bottom or U-bottom plates and stimulated with various concentrations of CDNs or IL-4 for the indicated time. The following stimuli were used: IL-4 (Supernatant from a J558L culture, 1:200 dilution); c-diGMP (C 057, Biolog life science institute, Federal Republic of Germany); 2′, 3′ cGAMP (c[G(2′,5′)pA(3′,5′)p], C 161, Biolog life science institute, Federal Republic of Germany); 3′, 3′ cGAMP (c-(ApGp), C 117, Biolog life science institute, Federal Republic of Germany); c-diAMP (C 088, Biolog life science institute, Federal Republic of Germany); Rp, Rp-c-diAMPSS (C118, Biolog life science institute, Federal Republic of Germany).

### Bone Marrow Derived Macrophage Stimulation

Bone Marrow Derived Macrophages (BMDM) were generated *in vitro* by flushing bone marrow from both femurs and tibias. Cells were cultured for 6 days in medium that induces the differentiation of macrophages (DMEM supplemented with 10% FBS, 1% sodium pyruvate, 1% L-glutamine, 1% penicillin/streptomycin, 2-mercaptoethanol, and 10% L-cell conditioned media). 5 mL of fresh media was added at day 3. At day 6 BMDMs were plated at 1 × 10^6^ cells in 1 ml in 12 well plates. The next day the cells were stimulated with 10 μg/ml CDG or 2′3′ cGAMP or 100 ng/ml LPS (Sigma, MO.) for 5 h. Supernatants were harvested and IFNβ was determined by ELISA (VeriKine-HS Mouse Interferon Beta Serum ELISA Kit, PBL Assay Science, NJ).

### Flow Cytometric Assay

Cells were harvested from cultures and incubated with optimal amounts of antibodies. For analysis of cell surface markers, cells were stained with antibodies directed against the following molecules: B220-BV421 (clone RA3-6B2, BD Bioscience, CA) and CD86-BV605 (clone GL1, BD biosciences, CA). Annexin V-FITC (BD Bioscience, CA) was used to identify dead/dying cells and only Annexin V^−^ cells were analyzed. FACS was performed using a Fortessa X-20 cytometer (BD biosciences, CA.) and analyzed using FlowJo software (Flowjo, Ashland, OR.).

### Western Blot Analysis

Mature B cells were purified by CD43 negative selection using MACS Miltenyi microbeads (Miltenyi Biotec Inc., San Diego, CA.) and lysed in 1% NP40 lysis buffer (1% Nonidet P-40, 150 mM NaCl, 10 mM Tris, pH 7.5,) supplemented with Protease/Phosphatase Inhibitor Cocktail (Cell Signaling, MA.) and 1 mM PMSF. Lysates of ~1 × 10^6^ cells were run in each lane of a 10% SDS-PAGE gel, and the proteins were transferred to PVDF membrane (GE/Amersham, PA.). The membrane was blotted with rabbit anti-mouse STING antibody (clone D2P2F, Cell Signaling, MA.) and goat anti-mouse Actin antibody (clone I-19, Santa Cruz Biotechnology, TX.) overnight followed by HRP-conjugated anti-rabbit IgG (Cell Signaling, MA.) and anti-goat IgG secondary antibodies (R&D Systems). The membrane was exposed to chemiluminescent substrate (SuperSignal West Pico Plus, ThermoScientific, MA.) and the chemiluminescence was detected using ChemiDoc XRS Imagining system (Bio-Rad Laboratories, CA.). The intensity of the bands was quantified using ImageStudioLite (Li-Cor Biosciences, NE).

### Immunization and Sample Collection

Mice were anesthetized with isofluorane in an E–Z anesthesia system (Euthanex, Palmer, PA.) and subjected to intranasal (i.n.) immunization. Mice received three bi-weekly immunizations administered by drop-wise 40 μL/nostril doses of endotoxin-low OVA (20 μg/ mouse) in PBS with or without CDN (5 μg/mouse ci-di-GMP or 5 μg/mouse 2′, 3′ cGAMP (c[G(2′,5′)pA(3′,5′)p], Biolog life science institute, Federal Republic of Germany). Endotoxin was removed (<0.1 EU) from OVA (Sigma, MO) as described (34) or commercial endotoxin-low OVA was used (Biovendor, Asheville, NC.). Serum was collected at the indicated time points and nasal passages were lavaged at the final time point with 1 mL of ice-cold PBS.

### Enzyme Linked Immunosorbent Assay (ELISA)

For detection of OVA-specific antibodies, microtiter plates were coated overnight with 10 μg /mL OVA in PBS at 4°C and blocked with 2% BSA in PBS 0.05% Tween-20 for >1 h at RT. Serial dilutions of mouse serum in PBS were added and incubated overnight at 4°C. IgG antibodies were detected with goat anti-mouse IgG-HRP (Southern Biotech, Birmingham, AL.) and IgA antibodies were detected with goat anti-mouse IgA-HRP(Southern Biotech, Birmingham, AL.) Between each step the plates were washed 4 times with PBS-0.05% Tween-20. The ELISA was developed with TMB single solution (Invitrogen, Carlsbad, CA.) and the reaction was stopped with 1N H_2_PO_4_ (Sigma-Aldrich, St. Louis, MO.). The OD was determined at 450 nm using a VERSAMax plate reader (Molecular Devices, Sunnyvale, CA.) and the data were analyzed with Softmax software (Molecular Devices, Sunnyvale, CA.). To plot the kinetic of antibody responses the half-max reciprocal of dilution was calculated by using the reciprocal of the dilution factor at a set half-max value for each group at each time point. In order to normalize results of assays performed on different days, a single serum sample collected on Day 42 from the WT mice that were immunized with OVA in the presence of CDG was run on each day.

### Statistical Analysis

The statistical significance was determined using the unpaired Student's *t*-test (Graphpad Prism 5.0; Graphpad, CA).

## Results

### Homozygous STING-HAQ B Cells and STING-R231H B Cells Do Not Respond to Exogenous CDNs *in vitro*

**Figure 1:**
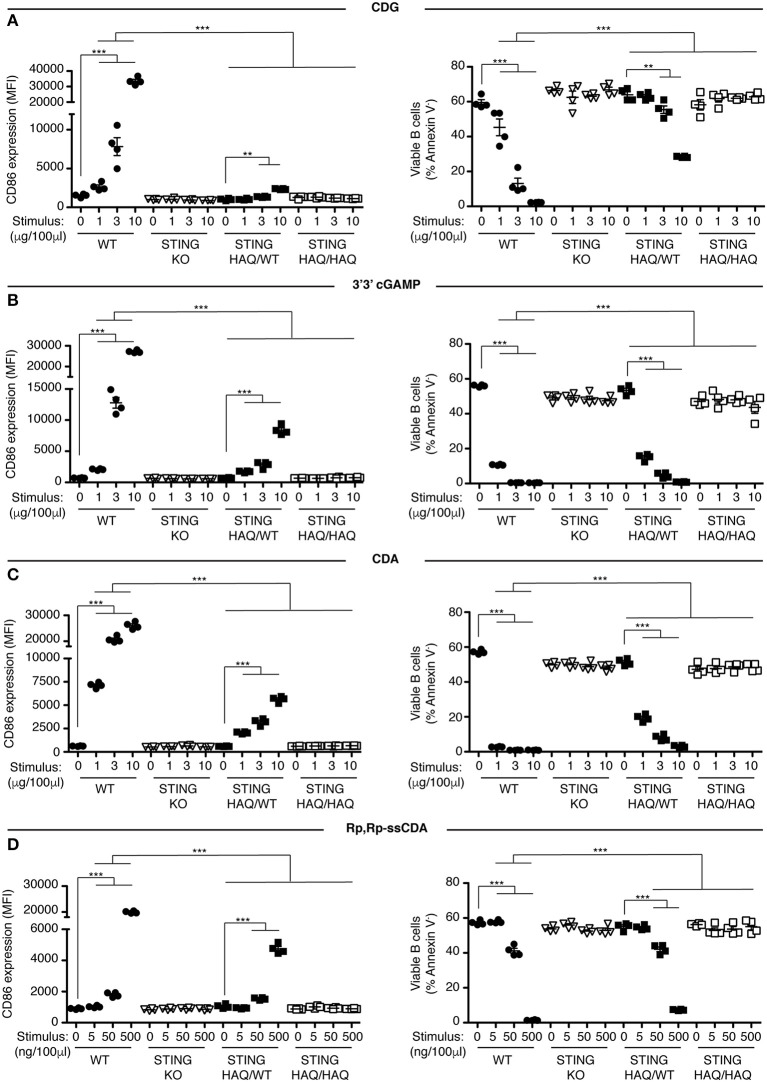
STING-HAQ B cells are impaired in their response to exogenous CDNs. **(A–D)** WT, STING KO, STING-HAQ/WT, and STING-HAQ/HAQ B cells (*n* = 4) were cultured for 18 h with the indicated concentrations of exogenous CDNs. CD86 expression (MFI gated on B220^+^ Annexin V^−^) is plotted in the left-hand column and B cell survival (% B220^+^ Annexin V^−^ cells) after stimulation is plotted in the right-hand column. B cells were stimulated with CDG **(A)**, 3′3′ cGAMP **(B)**, CDA **(C)**, and Rp,Rp-ssCDA **(D)**. Data shown are representative of at least two independent replicate experiments. Bars in **(A–D)** represent mean ± SEM. Statistical comparisons were made for each stimulus concentration and the range of concentrations that reached statistical significance between genotypes are indicated by horizontal lines above the graph. **P < 0.01; ***P < 0.001 using an unpaired Student's *t*-test.

**Figure 2:**
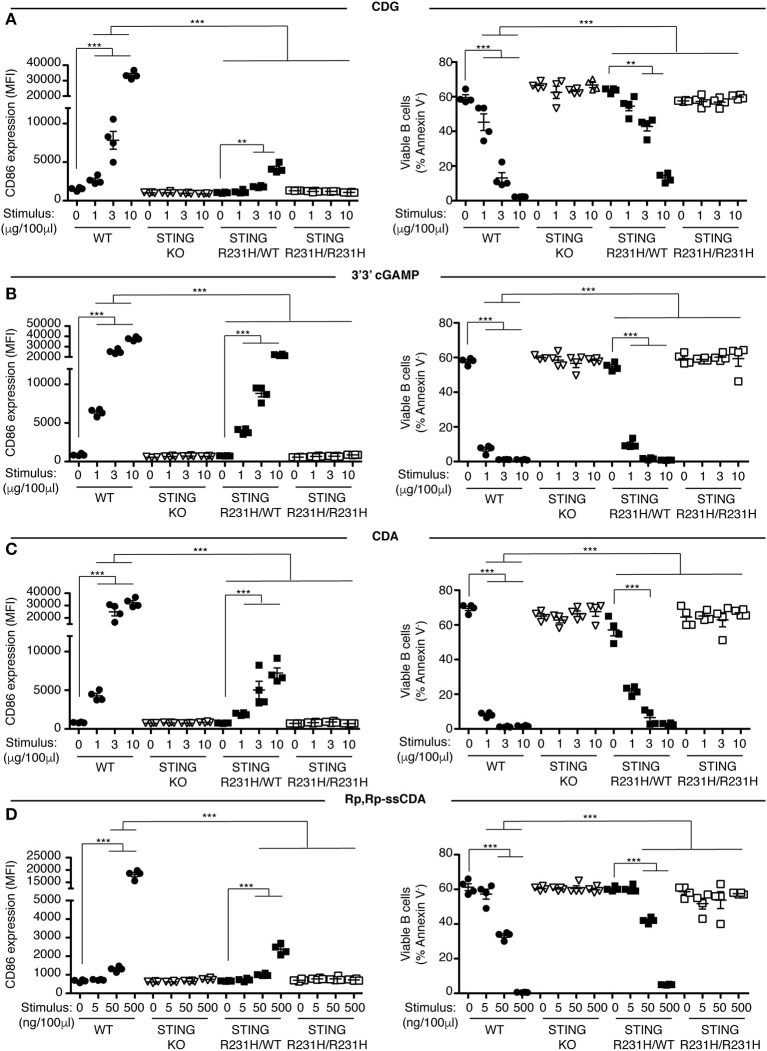
STING-R231H B cells are impaired in their response to exogenous CDNs. **(A–D)** WT, STING KO, STING-R231H/WT, and STING-R231H/R231H B cells (*n* = 4) were cultured for 18 h with the indicated concentrations of exogenous CDNs. CD86 expression (MFI gated on B220^+^ Annexin V^−^) is plotted in the left-hand column and B cell survival (% B220^+^ Annexin V^−^ cells) after stimulation is plotted in the right-hand column. B cells were stimulated with CDG **(A)**, 3′3′ cGAMP **(B)**, CDA **(C)**, and Rp,Rp-ssCDA **(D)**. Data shown are representative of at least two independent replicate experiments. Bars in **(A–D)** represent mean ± SEM. Statistical comparisons were made for each stimulus concentration and the range of concentrations that reached statistical significance between genotypes are indicated by horizontal lines above the graph. **P < 0.01; ***P < 0.001 using an unpaired Student's *t*-test.

**Figure 3:**
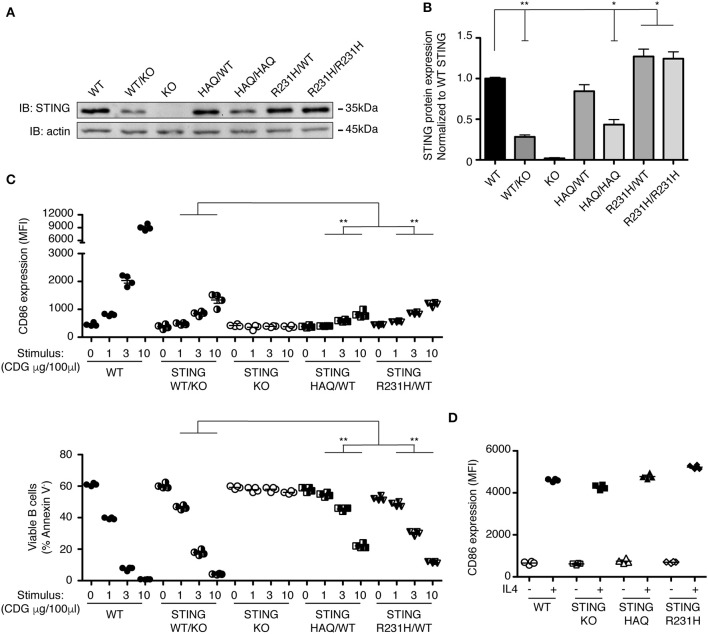
Reduced STING protein expression in STING-HAQ but not STING-R231H B cells. **(A,B)** STING protein expression in WT, STING^+/−^, STING^−/−^, STING-HAQ/WT, STING-HAQ/HAQ, STING-R231H/WT, and STING-R231H/R231H B cells. A representative blot is shown in **(A)**, normalized STING expression (*n* = 6/genotype) is shown in **(B)**. **(C)** WT, STING^+/−^, STING KO, STING-HAQ/WT, and STING-R231H/WT B cells (*n* = 4) were cultured for 18 h with the indicated concentrations of CDG. CD86 expression (MFI gated on B220^+^ Annexin V^−^) is plotted (top graph) and B cell survival (% B220^+^ Annexin V^−^ cells) after stimulation is plotted in the bottom graph. **(D)** WT, STING KO, STING-HAQ/HAQ, and STING-R231H/R231H B cells (*n* = 4) were cultured for 18 h with IL4 and CD86 expression (MFI) was measured. Data shown are representative of at least two independent replicate experiments. Bars in **(A–D)** represent mean ± SEM. Statistical comparisons were made for each stimulus concentration and the range of concentrations that reached statistical significance between genotypes are indicated by horizontal lines above the graph. *P < 0.05; **P < 0.01 using an unpaired Student's *t*-test.

Previously we showed that B cells respond *in vitro* to stimulation with CDNs by upregulating the costimulatory molecule CD86 and by undergoing caspase-dependent cell death (33). We used these parameters to assay the functional consequences of expressing STING-HAQ or STING-R231H variants on the ability of immune cells to respond to exogenous CDNs. Knock-in mice expressing these two common STING variants were generated in our laboratory. The STING-HAQ mouse has been described previously (29) and the generation of the STING-R231H mouse is described in Figure S1. The R231H point mutation in mice corresponds to the common allelic variant R232H described in humans. Purified B cells from homozygous STING-HAQ/HAQ (Figure 1) or homozygous STING-R231H/R231H (Figure 2) were cultured for 18 h with varied concentrations of the bacterial derived CDNs: cyclic-di-GMP (CDG), cyclic-di-AMP (CDA) and 3′,3′ cyclic-GMP–AMP (3′3′ cGAMP). In addition, B cells were cultured with a synthetic CDN: Rp.Rp-ssCDA, an adjuvant with increased stability and affinity for STING that is used in cancer immunotherapy (6). B cell expressing WT STING responded to these exogenous CDNs by upregulation of CD86 and cell death in a dose-dependent manner (Figures 1, 2, Figures S2–S4). This response is STING dependent as indicated by the absence of a response by deficient of STING (STING KO) B cells. In contrast, neither homozygous STING-HAQ/HAQ B cells (Figure 1) nor homozygous STING-R231H/R231H B cells (Figure 2) were able to respond to exogenous CDNs or to the synthetic CDN. This inability to respond to exogenous CDNs was not due to an inability of these B cells to become activated because both STING-HAQ/HAQ B cells and STING-R231H/R231H B cells respond to a STING-independent stimulus, IL4, by upregulating CD86 (Figure 3D).

### Heterozygous STING-HAQ B Cells and STING-R231H B Cells Have a Reduced Ability to Respond to Exogenous CDNs *in vitro*

Individuals that are homozygous for STING-HAQ or STING-R232H are relatively rare (2–3% of the population). Yet up to 20% of the population carries 1 copy of STING-HAQ and up to 13.7–17.9% of the population carries 1 copy of STING-R232H (27, 28). To test the consequence of STING-HAQ and STING-R231H heterozygosity on the ability of immune cells to respond to exogenous CDNs, we determined the ability of B cells isolated from heterozygous STING-HAQ or STING-R231H mice to respond to exogenous CDNs. Heterozygosity of both STING variants resulted in a greatly reduced (75–95% reduction in increase of CD86 MFI compared to STING WT) responses to exogenous CDNs (Figures 1, 2). The response to CDG was most affected by the presence of either STING-HAQ (Figure 1A) or STING-R231H (Figure 2A) whereas the response to 3′3′ cGAMP and CDA was less affected. The R231H and G230**A** (H**A**Q) point mutations both lie within one of the CDN binding regions of STING (35) so altered interactions with different CDNs could be expected.

A previous report described reduced STING expression in human and murine tissues expressing STING-HAQ (29). We confirmed these findings in B cells from STING-HAQ mice, detecting a ~50% reduction of detectable STING protein in B cells from homozygous STING-HAQ mice and a ~20% reduction in B cells from heterozygous STING-HAQ (Figures 3A,B). No reduction of STING expression was found in B cells homozygous for STING-R231H (Figures 3A,B), in fact these mice appeared to have slightly higher STING protein expression.

STING forms homodimers that undergo a conformational change upon CDN binding, leading to oligomerization, recruitment and activation of tank-binding kinase 1 (TBK1), that in turn activates IRF3 (36, 37). This raises the question whether in individuals that are heterozygous for either STING-HAQ or STING-R231H, the point-mutated STING acts in a dominant negative fashion when forming heterodimers with WT STING. To approach this question, we compared the response of STING-HAQ or STING-R231H heterozygous B cells to exogenous CDNs, to that of STING haploinsufficient B cells. STING haploinsuffient B cells express ~30% of STING protein compared to WT STING B cells (Figure 3B). Despite having lower STING protein levels than heterozygous STING-HAQ or STING-R231H mice, STING haploinsufficient mice have stronger responses to CDG (Figure 3C, Figure S5) and CDA (data not shown). Assuming equal expression of both WT and mutant STING and a dominant negative function of the mutant STING, only 25% of available STING in heterozygous STING-HAQ and STING-R231H B cells would be STING-WT/WT dimers and functional. The observed reduced ability of STING-HAQ and STING-R231H heterozygous B cells to respond to exogenous CDNs, while homozygous STING-HAQ and STING-R231H B cells are unable to respond (Figures 1, 2) is consistent with ~25% WT homodimers present responsible for the observed response. These results are suggestive of a dominant negative effect of the HAQ and R231H STING variants expression on WT STING function, as has been described for other STING variants (17).

### Immune Cells Homozygous for STING-R231H but Not STING-HAQ Respond to Endogenous 2′3′ cGAMP

**Figure 4:**
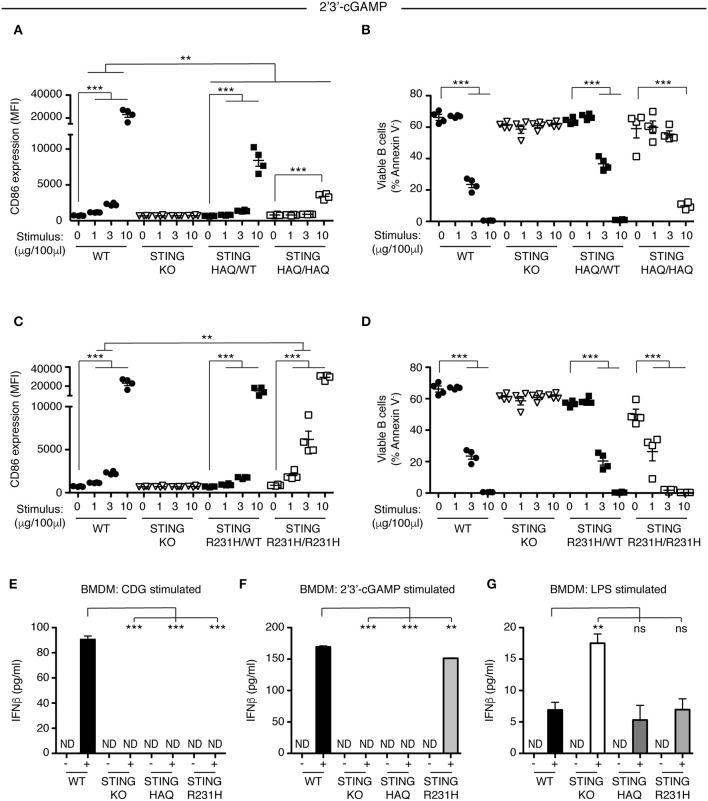
STING-HAQ expressing cells but not STING-R231H expressing cells are impaired in their response to endogenous 2′3′ cGAMP. **(A,B)** WT, STING KO, STING-HAQ/WT, and STING-HAQ/HAQ B cells (*n* = 4) were cultured for 18 h with the indicated concentrations of 2′3′ cGAMP. **(A)** CD86 expression (MFI, gated on B220^+^ Annexin V^−^) and **(B)** survival (% Annexin V^−^) of B cells stimulated with 2′3′ cGAMP. **(C,D)** WT, STING KO, STING-R231H/WT, and STING-R231H/R231H B cells (*n* = 4) were cultured for 18 h with the indicated concentrations of 2′3′ cGAMP. **(C)** CD86 expression (MFI, gated on B220^+^ Annexin V^−^) and **(D)** survival (% Annexin V^−^) of B cells stimulated with 2′3′ cGAMP. **(E–G)** Bone-marrow derived macrophages generated (*n* = 3) from WT, STING KO, STING-HAQ/HAQ, and STING-R231H/R231H mice were incubated with 10 μg/ml CDG **(E)**, 10 μg/ml 2′3′ cGAMP **(F)** or 100 ng/ml LPS **(G)** for 5 h and the concentration of IFNβ in the supernatant was measured by ELISA. Data shown are representative of at least two independent replicate experiments. Bars in **(A–D)** represent mean ± SEM. Statistical comparisons were made for each stimulus concentration and the range of concentrations that reached statistical significance between genotypes are indicated by horizontal lines above the graph. ND: not detected, **P < 0.01; ***P < 0.001 using an unpaired Student's *t*-test.

STING plays a central role in intracellular DNA sensing pathways by binding the cGAS product 2′3′ cGAMP, resulting in IRF3 activation. Previous work suggested that STING-HAQ is a null allele, disabling the carrier's ability to respond to 2′3′ cGAMP (29), although others have reported residual ability of PBMCs of STING-HAQ/HAQ carriers to respond to intracellular DNA or cGAMP (38, 39). We incubated heterozygous and homozygous STING-HAQ B cells with increasing concentrations of 2′3′ cGAMP. B cells from STING-HAQ/WT mice have reduced ability to respond to 2′3′ cGAMP at all concentrations tested (Figures 4A,B, Figure S6). Homozygous STING-HAQ B cells did not respond to the lower doses of 2′3′ cGAMP. However, we reproducibly observed that at the highest concentration of 2′3′ cGAMP tested STING-HAQ/HAQ B cells do respond to 2′3′ cGAMP by upregulation of CD86 (Figure 4A) and increased cell death (Figure 4B). This suggests that STING-HAQ is not a null allele, but rather is a hypomorph with greatly reduced ability to respond to 2′3′ cGAMP.

Next, we analyzed the ability of STING-R231H B cells to respond to 2′3′ cGAMP. Data from ectopic expression experiments suggest that this STING variant can still respond to endogenous CDN (27, 28). We stimulated STING-R231H homozygous and heterozygous B cells with different concentrations of 2′3′ cGAMP. In contrast to their inability to respond to exogenous CDNs (Figure 2), STING-R231H/R231H B cells are fully responsive to 2′3′ cGAMP (Figures 4C,D). In fact, they respond better, although this could be due to slightly increased STING expression (Figure 3A).

To verify that the observed responsiveness to CDNs in B cells is not cell type specific, we generated bone-marrow derived macrophages (BMDM) from mice homozygous for STING-HAQ and STING-R231H and tested their ability to respond to exogenous and endogenous CDNs (Figures 4E,F). While WT BMDMs responded to CDG (Figure 4E) and 2′3′ cGAMP (Figure 4F) by producing INFβ, homozygous STING-HAQ BMDMs were impaired in their response to both CDG and 2′3′ cGAMP. In contrast homozygous STING-R231H BMDMs only responded to 2′3′ cGAMP but not CDG. These results confirm our findings using B cells (Figures 1A, 2A, 4A, 4C). All genotypes were able to respond to LPS by producing INFβ (Figure 4G), demonstrating that the inability to respond to CDNs does not reflect a general defect in INFβ responses. The enhanced response of STING KO BMDM to LPS was not a reproducible finding.

Together these results show that *ex vivo* immune cells expressing STING-HAQ are greatly impaired in their response to both exogenous and endogenous CDNs, while immune cells expressing STING-R231H are impaired in their ability to respond to exogenous CDN but not endogenous CDNs.

### STING-HAQ Mice Do Not Respond to CDN Adjuvants

**Figure 5:**
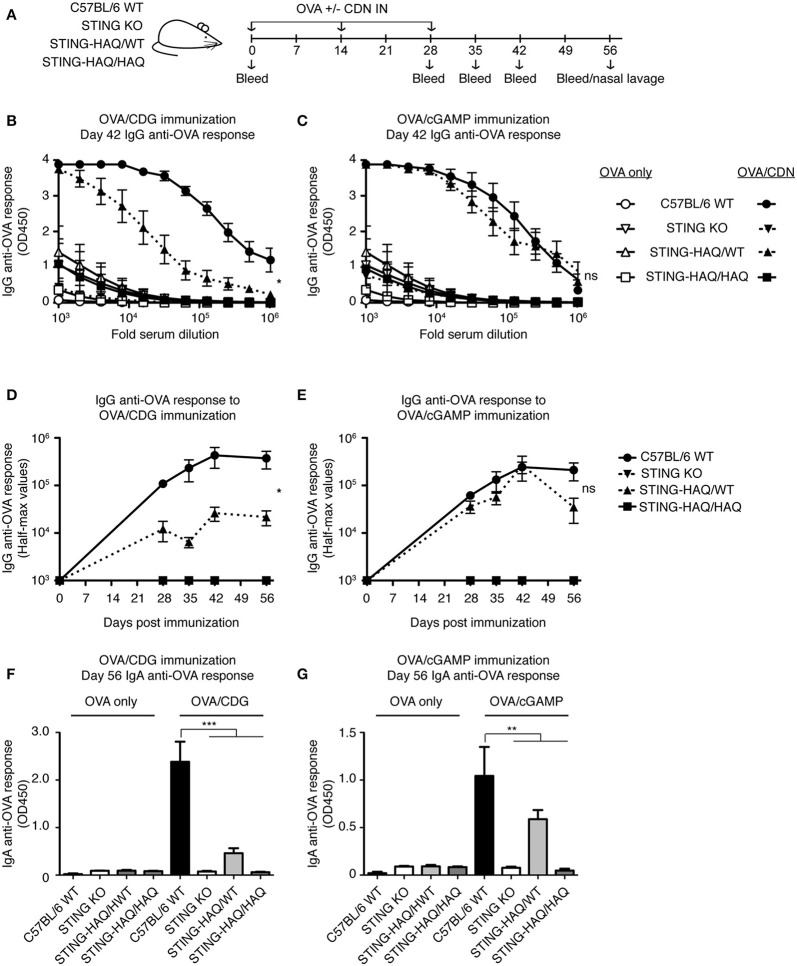
STING-HAQ homozygous mice are unresponsive to immunization with CDN adjuvant. **(A–G)** WT, STING KO and STING-HAQ/WT, and STING-HAQ/HAQ were immunized IN with 20 μg OVA with or without 5 μg CDG **(B,D,F)** or 5 μg 2′3′ cGAMP **(C,E,G)**, and serum and mucosal anti-OVA antibody responses were monitored (*n* = 5). **(A)** Schematic representation of the experiment; **(B,C)** IgG anti-OVA response at d42 post-immunization, **(D,E)** Reciprocal half-max values of the OVA-specific IgG response; **(F,G)** OVA-specific IgA response in nasal wash at day 56. Data shown are representative of at least two independent replicate experiments. Bars in **(B–G)** represent mean ± SEM. NS *P* > 0.05; *P < 0.05; **P < 0.01; ***P < 0.001 using an unpaired Student's *t*-test.

To determine whether these observations hold true *in vivo* we examined the ability of STING-HAQ and STING-R231H mice to respond to immunization when CDNs are used as adjuvants. CDG adjuvant activity is well-defined and requires STING expression in multiple cell types, including CD11c^+^ dendritic cells (40, 41) and B cells (33), to promote optimal antibody responses. 2′3′ cGAMP has also been used as an adjuvant (29) but can also act as a proxy for *in vivo* responses that elicit a cGAS-mediated response. STING-HAQ homozygous and heterozygous mice were immunized with OVA in the presence or absence of CDG or 2′3′ cGAMP (Figure 5A). As shown previously (29, 33, 42) mice expressing WT STING mount greatly enhanced anti-OVA responses when CDG (Figures 5B,D,F) or 2′3′ cGAMP (Figures 5C,E,H) is used as an adjuvant. These responses to CDN adjuvants are STING-dependent, as they are absent in STING deficient animals. In agreement with the observed *in vitro* responses to CDG (Figure 1A), immunization using CDG as an adjuvant did not result in enhanced titers of serum anti-OVA IgG (Figures 5B,D) or mucosal anti-OVA IgA (Figure 5F) in STING-HAQ/HAQ mice. Heterozygous STING-HAQ/WT were able to respond to CDG adjuvants, albeit with reduced magnitude. Similarly, the adjuvant effect of 2′3′ cGAMP was lost in STING-HAQ/HAQ mice (Figures 5C,E,G), while STING-HAQ/WT mice were only marginally impaired in their ability to respond to 2′3′ cGAMP.

### STING-R231H Mice Are Selectively Impaired in Their Response to Exogenous CDG *in vivo*, While Retaining Their Ability to Respond to Endogenous 2′3′ cGAMP

**Figure 6:**
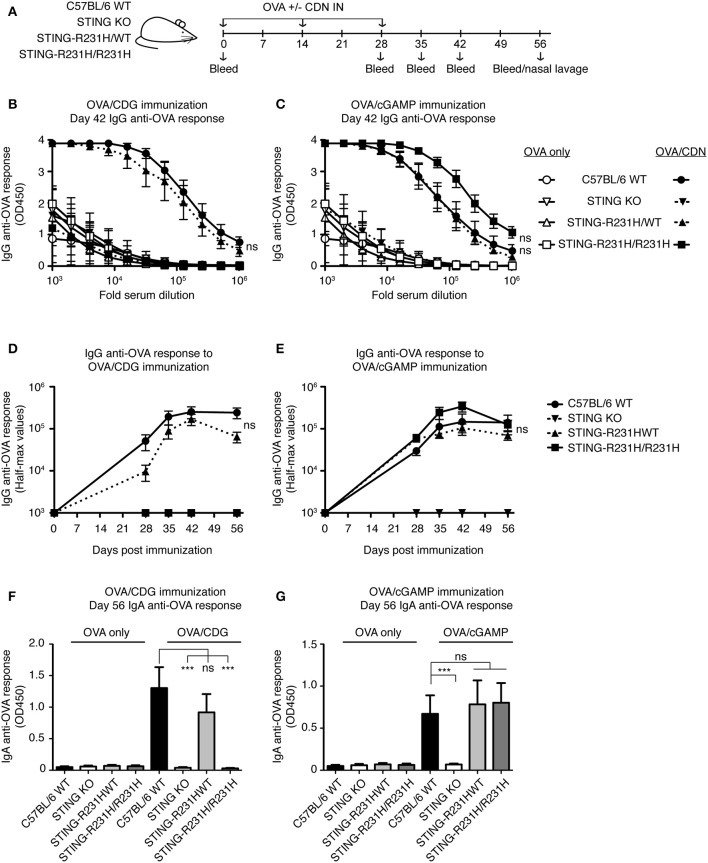
STING-R231H homozygous mice are unresponsive to immunization using CDG as an adjuvant but remain responsive to immunization using 2′3′ cGAMP as an adjuvant. **(A–G)** WT, STING KO and STING-R231H/WT and STING-R231H/R231H were immunized IN with 20 μg OVA with or without 5 μg CDG **(B,D,F)** or 5 μg 2′3′ cGAMP **(C,E,G)**, and serum and mucosal anti-OVA antibody responses were monitored (*n* = 5). **(A)** Schematic representation of the experiment; **(B,C)** IgG anti-OVA response at d42 post-immunization, **(D,E)** Reciprocal half-max values of the OVA-specific IgG response; **(F,G)** OVA-specific IgA response in nasal wash at day 56. Data shown are representative of at least two independent replicate experiments. Bars in B-G represent mean ± SEM. NS, *P* > 0.05; ***P < 0.001 using an unpaired Student's *t*-test.

To test the *in vivo* consequences of STING-R231H expression, we immunized mice homozygous or heterozygous for STING-R231H with OVA in the presence of absence of CDG or 2′3′ cGAMP (Figure 6A). As observed *in vitro* (Figure 2A), STING-R231H/R231H mice did not mount enhanced anti-OVA IgG (Figures 6B,D) or IgA (Figure 6F) responses following immunization using CDG as an adjuvant. In contrast, heterozygous STING-R231H/WT mouse responses to CDG/OVA were only marginally reduced compared to STING WT mice. The response of STING-R231H/WT mice to 2′3′ cGAMP/OVA immunization was of the same magnitude as observed in STING WT mice (Figures 6 C,E,G). Homozygous STING-R231H/R231H mice trended toward enhanced anti-OVA IgG response following 2′3′ cGAMP/OVA immunization, compared to STING-WT mice (Figures 6C,E).

Taken together these data confirm and extend previous reports that expression of STING-HAQ compromises the ability of carriers to respond to exogenous and endogenous CDNs, while expression of STING-R231H compromises the ability to respond to exogenous CDNs while it does not affect the cGAS/STING pathway.

## Discussion

In this report we describe two novel mouse models, representing the two most prevalent minor STING alleles, and their ability to respond to exogenous and endogenous CDNs *in vitro* and *in vivo*. Under the conditions tested, the STING-HAQ allele conferred unresponsiveness to all exogenous CDNs tested and caused severely reduced responsiveness to endogenous 2′3′ cGAMP (Figures 1, 3–5). In contrast, the STING-R231H allele (murine equivalent to human R232H) conferred responsiveness to endogenous 2′3′ cGAMP, but disabled responses to the exogenous CDNs tested (Figures 2–4, 6).

The STING-HAQ function was initially characterized by Patel et al. (29) in a study that was limited to analysis to homozygous STING-HAQ mice and reported that the allele was a null allele, lacking responsiveness to both endogenous and exogenous CDNs. Analysis of human EBV immortalized STING-HAQ B cells supported these findings. Our analysis of the STING-HAQ mice confirmed the finding of Patel et al. (Figures 1, 5) and expand them to show that STING-HAQ heterozygosity impacts the ability to respond to CDNs as well *in vitro* and *in vivo* (Figures 1, 3, 5). A noteworthy difference is that in our hands homozygous STING-HAQ retains some responsiveness to high concentrations of 2′3′ cGAMP (Figures 4A,B), indicating that STING-HAQ is not functionally null, but rather has reduced affinity for 2′3′ cGAMP resulting in near absent responses. These data agree with observations made using PBMCs from STING-HAQ/HAQ individuals, which show a strongly reduced but not absent responses to 2′3′ cGAMP and DNA (38). It should be noted that others have reported that STING-HAQ PBMCs are responsive to both exogenous and endogenous CDNs (43). The reason for this discrepancy is unclear.

The selective unresponsiveness to exogenous CDNs but not to 2′3′ cGAMP observed in STING R231H mice is in agreement with previous studies of ectopically expressed R232H or *R231A* STING variants (4, 7, 27, 28). The observed *in vivo* responses in STING-R231H mice are likely to accurately reflect responsiveness of individuals homozygous for STING-R232H. PBMCs from individuals that are STING-R232H homozygous also have an impaired responsiveness to CDG, but respond normally to 2′3′ cGAMP and DNA (38). It is noteworthy that EBV-immortalized B cells from R232H homozygous individuals are severely impaired in their ability to respond to exogenous CDNs and 2′3′ cGAMP stimulation (29). The reason for this disparity is unclear, however, it is possible that immortalization using DNA virus infection led to selective outgrowth of subclones with decreased responsiveness to 2′3′ cGAMP.

The STING-HAQ and STING-R231H mice described here should be valuable tools to examine the effects of the respective human alleles on the susceptibility to infections and responsiveness to CDN-based vaccines and therapeutics. The first reports examining prevalence of STING-HAQ or STING-R232H variants among patients infected with *Legionella pneumophilia* (38) and *Streptococcus pneumoniae* (39) paint a complicated picture. While both pathogens are detected in a STING-dependent manner *in vitro*, an increased frequency among patients in STING-HAQ heterozygous and homozygous individuals was only observed among patients with Legionnaires' disease. This differential *in vivo* dependence of functional cGAS/STING to control infections was confirmed using knockout mice. In neither study were differences in R232H prevalence reported. Another study suggests that STING-HAQ homozygosity may result in slower disease progression in HIV-infected individuals (44). Systematic studies using mouse models such as the ones described in this manuscript could be useful in predicting clinical consequences of carrying these genetic variants.

STING plays a role in several sensing pathways making it hard to dissect the role STING plays in individual pathways. An example is found in the fact that during bacterial infections STING can be activated by multiple mechanisms. It acts as a sensor of bacterial derived CDNs directly, independent of cGAS (14, 45, 46), and may sense other bacterial products (47) in a cGAS independent manner. STING also plays a central role in detection of DNA from bacteria and infection-induced host cell damage by sensing 2′3′ cGAMP produced by the DNA sensor cGAS (14). Adding to the complexity, in the immune response to *Brucella abortus* both IFN and IL-1 responses are STING-dependent but only the IFN response is cGAS-dependent (48). To gain insight into the different mechanisms by which STING contributes to immune responses, investigators often compare STING KO mice to cGAS KO mice. However, such an approach has limitations. For example, cGAS has been proposed to enhance the response to extracellular CDNs by binding endocytosed CDNs and presenting them to STING (49). Other studies suggest that cGAS may have additional functions independent of STING (50) that could confound findings. The STING-R231H/R231H mutant mouse circumvents these issues by retaining responsiveness to cGAS-derived CDN (Figures 4, 6), while being unable to respond to exogenous CDNs (Figures 2, 6), eliminating potentially confounding effects of cGAS deficiency. Such an approach has been used successfully *in vitro* in the context of *Chlamydia trachomatis* infection, by ectopic expression of a R231A mutant (51). In this respect the R231H mouse model could be a valuable tool in the arsenal of weapons available to examine the role STING-mediated anti-DNA responses vs. exogenous CDN responses, in the context of bacterial infections or CDN-based anti-tumor responses.

## Data Availability Statement

Representative datasets generated for this study are included in the article/Supplementary Material. The raw data supporting the conclusions of this article will be made available by the authors, without undue reservation, to any qualified researcher.

## Ethics Statement

The animal study was reviewed and approved by University of Colorado and National Jewish Health Institutional Animal Care and Use Committee.

## Author Contributions

MW, SK, WC, KN, and AG performed the experiments. MW, SK, and AG analyzed the data. MW, LL, JC, and AG designed the experiments and wrote the paper. All authors read and approved the submitted version.

### Conflict of Interest

The authors declare that the research was conducted in the absence of any commercial or financial relationships that could be construed as a potential conflict of interest.

## References

[1] Jin L Waterman PM Jonscher KR Short CM Reisdorph NA Cambier JC. MPYS, a novel membrane tetraspanner, is associated with major histocompatibility complex class II and mediates transduction of apoptotic signals. Mol Cell Biol. (2008) 28:5014–26. 10.1128/MCB.00640-08 18559423 PMC2519703

[2] Ishikawa H Barber GN. STING is an endoplasmic reticulum adaptor that facilitates innate immune signalling. Nature. (2008) 455:674–8. 10.1038/nature07317 18724357 PMC2804933

[3] Zhong B Yang Y Li S Wang YY Li Y Diao F. The adaptor protein MITA links virus-sensing receptors to IRF3 transcription factor activation. Immunity. (2008) 29:538–50. 10.1016/j.immuni.2008.09.003 18818105

[4] Burdette DL Monroe KM Sotelo-Troha K Iwig JS Eckert B Hyodo M. STING is a direct innate immune sensor of cyclic di-GMP. Nature. (2011) 478:515–8. 10.1038/nature10429 21947006 PMC3203314

[5] Sauer JD Sotelo-Troha K von Moltke J Monroe KM Rae CS Brubaker SW. The N-ethyl-N-nitrosourea-induced Goldenticket mouse mutant reveals an essential function of Sting in the *in vivo* interferon response to Listeria monocytogenes and cyclic dinucleotides. Infect Immun. (2011) 79:688–94. 10.1128/IAI.00999-10 21098106 PMC3028833

[6] Fu J Kanne DB Leong M Glickman LH McWhirter SM Lemmens E. STING agonist formulated cancer vaccines can cure established tumors resistant to PD-1 blockade. Sci Transl Med. (2015) 7:283ra52. 10.1126/scitranslmed.aaa4306 25877890 PMC4504692

[7] Diner EJ Burdette DL Wilson SC Monroe KM Kellenberger CA Hyodo M. The innate immune DNA sensor cGAS produces a noncanonical cyclic dinucleotide that activates human STING. Cell Rep. (2013) 3:1355–61. 10.1016/j.celrep.2013.05.009 23707065 PMC3706192

[8] Wu J Sun L Chen X Du F Shi H Chen C. Cyclic GMP-AMP is an endogenous second messenger in innate immune signaling by cytosolic DNA. Science. (2013) 339:826–30. 10.1126/science.1229963 23258412 PMC3855410

[9] Dunphy G Flannery SM Almine JF Connolly DJ Paulus C Jonsson KL. Non-canonical activation of the DNA sensing adaptor STING by ATM and IFI16 mediates NF-κB signaling after nuclear DNA damage. Mol Cell. (2018) 71:745–60 e5. 10.1016/j.molcel.2018.07.034 30193098 PMC6127031

[10] Liu D Wu H Wang C Li Y Tian H Siraj S. STING directly activates autophagy to tune the innate immune response. Cell Death Differ. (2018) 26:1735–49. 10.1038/s41418-018-0251-z 30568238 PMC6748081

[11] Gui X Yang H Li T Tan X Shi P Li M. Autophagy induction via STING trafficking is a primordial function of the cGAS pathway. Nature. (2019) 567:262–6. 10.1038/s41586-019-1006-9 30842662 PMC9417302

[12] Watson RO Manzanillo PS Cox JS. Extracellular M. tuberculosis DNA targets bacteria for autophagy by activating the host DNA-sensing pathway. Cell. (2012) 150:803–15. 10.1016/j.cell.2012.06.040 22901810 PMC3708656

[13] Ishikawa H Ma Z Barber GN STING regulates intracellular DNA-mediated type I interferon-dependent innate immunity. Nature. (2009) 461:788–92. 10.1038/nature08476 19776740 PMC4664154

[14] Dey B Dey RJ Cheung LS Pokkali S Guo H Lee JH. A bacterial cyclic dinucleotide activates the cytosolic surveillance pathway and mediates innate resistance to tuberculosis. Nat Med. (2015) 21:401–6. 10.1038/nm.3813 25730264 PMC4390473

[15] Ahn J Gutman D Saijo S Barber GN. STING manifests self DNA-dependent inflammatory disease. Proc Natl Acad Sci USA. (2012) 109:19386–91. 10.1073/pnas.1215006109 23132945 PMC3511090

[16] Gall A Treuting P Elkon KB Loo YM Gale M Jr Barber GN. Autoimmunity initiates in nonhematopoietic cells and progresses via lymphocytes in an interferon-dependent autoimmune disease. Immunity. (2012) 36:120–31. 10.1016/j.immuni.2011.11.018 22284419 PMC3269499

[17] Konno H Yamauchi S Berglund A Putney RM Mule JJ Barber GN. Suppression of STING signaling through epigenetic silencing and missense mutation impedes DNA damage mediated cytokine production. Oncogene. (2018) 37:2037–51. 10.1038/s41388-017-0120-0 29367762 PMC6029885

[18] Woo SR Fuertes MB Corrales L Spranger S Furdyna MJ Leung MY. STING-dependent cytosolic DNA sensing mediates innate immune recognition of immunogenic tumors. Immunity. (2014) 41:830–42. 10.1016/j.immuni.2014.10.017 25517615 PMC4384884

[19] Kumar V. A STING to inflammation and autoimmunity. J Leukoc Biol. (2019) 106:171–85. 10.1002/JLB.4MIR1018-397RR 30990921

[20] Li T Chen ZJ. The cGAS-cGAMP-STING pathway connects DNA damage to inflammation, senescence, and cancer. J Exp Med. (2018) 215:1287–99. 10.1084/jem.20180139 29622565 PMC5940270

[21] Ebensen T Libanova R Schulze K Yevsa T Morr M Guzman CA. Bis-(3',5')-cyclic dimeric adenosine monophosphate: strong Th1/Th2/Th17 promoting mucosal adjuvant. Vaccine. (2011) 29:5210–20. 10.1016/j.vaccine.2011.05.026 21619907

[22] Hu DL Narita K Hyodo M Hayakawa Y Nakane A Karaolis DK. c-di-GMP as a vaccine adjuvant enhances protection against systemic methicillin-resistant Staphylococcus aureus (MRSA) infection. Vaccine. (2009) 27:4867–73. 10.1016/j.vaccine.2009.04.053 19406185

[23] Madhun AS Haaheim LR Nilsen MV Cox RJ. Intramuscular matrix-M-adjuvanted virosomal H5N1 vaccine induces high frequencies of multifunctional Th1 CD4+ cells and strong antibody responses in mice. Vaccine. (2009) 27:7367–76. 10.1016/j.vaccine.2009.09.044 19781678

[24] Gutjahr A Papagno L Nicoli F Kanuma T Kuse N Cabral-Piccin MP. The STING ligand cGAMP potentiates the efficacy of vaccine-induced CD8+ T cells. JCI Insight. (2019) 4. 10.1172/jci.insight.125107 30944257 PMC6483644

[25] Corrales L Glickman LH McWhirter SM Kanne DB Sivick KE Katibah GE. Direct activation of STING in the tumor microenvironment leads to potent and systemic tumor regression and immunity. Cell Rep. (2015) 11:1018–30. 10.1016/j.celrep.2015.04.031 25959818 PMC4440852

[26] Patel S Jin L. TMEM173 variants and potential importance to human biology and disease. Genes Immun. (2019) 20:82–9. 10.1038/s41435-018-0029-9 29728611 PMC6212339

[27] Yi G Brendel VP Shu C Li P Palanathan S Cheng Kao C. Single nucleotide polymorphisms of human STING can affect innate immune response to cyclic dinucleotides. PLoS ONE. (2013) 8:e77846. 10.1371/journal.pone.0077846 24204993 PMC3804601

[28] Jin L Xu LG Yang IV Davidson EJ Schwartz DA Wurfel MM. Identification and characterization of a loss-of-function human MPYS variant. Genes Immun. (2011) 12:263–9. 10.1038/gene.2010.75 21248775 PMC3107388

[29] Patel S Blaauboer SM Tucker HR Mansouri S Ruiz-Moreno JS Hamann L The common R71H-G230A-R293Q human TMEM173 is a null allele. J Immunol. (2017) 198:776–87. 10.4049/jimmunol.1601585 27927967 PMC5225030

[30] Zhang X Shi H Wu J Zhang X Sun L Chen C Cyclic GMP-AMP containing mixed phosphodiester linkages is an endogenous high-affinity ligand for STING. *Mol Cell*. (2013) 51:226–35. 10.1016/j.molcel.2013.05.022 PMC3808999 23747010

[31] Shi H Wu J Chen ZJ Chen C. Molecular basis for the specific recognition of the metazoan cyclic GMP-AMP by the innate immune adaptor protein STING. Proc Natl Acad Sci USA. (2015) 112:8947–52. 10.1073/pnas.1507317112 26150511 PMC4517257

[32] Jin L Hill KK Filak H Mogan J Knowles H Zhang B. MPYS is required for IFN response factor 3 activation and type I IFN production in the response of cultured phagocytes to bacterial second messengers cyclic-di-AMP and cyclic-di-GMP. J Immunol. (2011) 187:2595–601. 10.4049/jimmunol.1100088 21813776 PMC3159690

[33] Walker MM Crute BW Cambier JC Getahun A. B Cell-Intrinsic STING signaling triggers cell activation, synergizes with B cell receptor signals, and promotes antibody responses. J Immunol. (2018) 201:2641–53. 10.4049/jimmunol.1701405 30282750 PMC6497520

[34] Aida Y Pabst MJ. Removal of endotoxin from protein solutions by phase separation using Triton X-114. J Immunol Methods. (1990) 132:191–5. 10.1016/0022-1759(90)90029-U 2170533

[35] Martin M Hiroyasu A Guzman RM Roberts SA Goodman AG. Analysis of drosophila STING reveals an evolutionarily conserved antimicrobial function. Cell Rep. (2018) 23:3537–50.e6. 10.1016/j.celrep.2018.05.029 29924997 PMC6114933

[36] Zhang C Shang G Gui X Zhang X Bai XC Chen ZJ Structural basis of STING binding with and phosphorylation by TBK1. Nature. (2019) 567:394–8. 10.1038/s41586-019-1000-2 30842653 PMC6862768

[37] Shang G Zhang C Chen ZJ Bai XC Zhang X Cryo-EM structures of STING reveal its mechanism of activation by cyclic GMP-AMP. Nature. (2019) 567:389–93. 10.1038/s41586-019-0998-5 30842659 PMC6859894

[38] Ruiz-Moreno JS Hamann L Shah JA Verbon A Mockenhaupt FP Puzianowska-Kuznicka M The common HAQ STING variant impairs cGAS-dependent antibacterial responses and is associated with susceptibility to Legionnaires' disease in humans. PLoS Pathog. (2018) 14:e1006829 10.1371/journal.ppat.1006829 29298342 PMC5770077

[39] Ruiz-Moreno JS Hamann L Jin L Sander LE Puzianowska-Kuznicka M Cambier J The cGAS/STING pathway detects streptococcus pneumoniae but appears dispensable for antipneumococcal defense in mice and humans. Infect Immun. (2018) 86:e00849–17. 10.1128/IAI.00849-17 29263110 PMC5820968

[40] Blaauboer SM Mansouri S Tucker HR Wang HL Gabrielle VD Jin L. The mucosal adjuvant cyclic di-GMP enhances antigen uptake and selectively activates pinocytosis-efficient cells *in vivo*. Elife. (2015) 4:e06670. 10.7554/eLife.06670 25898005 PMC4428110

[41] Mansouri S Patel S Katikaneni DS Blaauboer SM Wang W Schattgen S. Immature lung TNFR2(-) conventional DC 2 subpopulation activates moDCs to promote cyclic di-GMP mucosal adjuvant responses *in vivo*. Mucosal Immunol. (2019) 12:277–89. 10.1038/s41385-018-0098-0 30327534 PMC6301145

[42] Blaauboer SM Gabrielle VD Jin L MPYS/STING-mediated TNF-α, not type I IFN, is essential for the mucosal adjuvant activity of (3′-5′)-cyclic-di-guanosine-monophosphate *in vivo*. J Immunol. (2014) 192:492–502. 10.4049/jimmunol.1301812 24307739 PMC6195764

[43] Sivick KE Surh NH Desbien AL Grewal EP Katibah GE McWhirter SM. Comment on The common R71H-G230A-R293Q human TMEM173 is a null allele. J Immunol. (2017) 198:4183–5. 10.4049/jimmunol.1700294 28533276

[44] Nissen SK Pedersen JG Helleberg M Kjaer K Thavachelvam K Obel N. Multiple homozygous variants in the STING-encoding TMEM173 Gene in HIV long-term nonprogressors. J Immunol. (2018) 200:3372–82. 10.4049/jimmunol.1701284 29632140

[45] Watson RO Bell SL MacDuff DA Kimmey JM Diner EJ Olivas J. The cytosolic sensor cGAS detects *Mycobacterium tuberculosis* DNA to induce type I interferons and activate autophagy. Cell Host Microbe. (2015) 17:811–9. 10.1016/j.chom.2015.05.004 26048136 PMC4466081

[46] Webster SJ Brode S Ellis L Fitzmaurice TJ Elder MJ Gekara NO. Detection of a microbial metabolite by STING regulates inflammasome activation in response to Chlamydia trachomatis infection. PLoS Pathog. (2017) 13:e1006383. 10.1371/journal.ppat.1006383 28570638 PMC5453623

[47] Movert E Lienard J Valfridsson C Nordstrom T Johansson-Lindbom B Carlsson F. Streptococcal M protein promotes IL-10 production by cGAS-independent activation of the STING signaling pathway. PLoS Pathog. (2018) 14:e1006969. 10.1371/journal.ppat.1006969 29579113 PMC5886698

[48] Costa Franco MM Marim F Guimaraes ES Assis RGN Cerqueira DM Alves-Silva J. Brucella abortus triggers a cGAS-independent STING pathway to induce host protection that involves guanylate-binding proteins and inflammasome activation. J Immunol. (2018) 200:607–22. 10.4049/jimmunol.1700725 29203515 PMC5760291

[49] Liu H Moura-Alves P Pei G Mollenkopf HJ Hurwitz R Wu X. cGAS facilitates sensing of extracellular cyclic dinucleotides to activate innate immunity. EMBO Rep. (2019) 20:e46293. 10.15252/embr.201846293 30872316 PMC6446192

[50] Yang H Wang H Ren J Chen Q Chen ZJ. cGAS is essential for cellular senescence. Proc Natl Acad Sci USA. (2017) 114:E4612–20. 10.1073/pnas.1705499114 28533362 PMC5468617

[51] Barker JR Koestler BJ Carpenter VK Burdette DL Waters CM Vance RE. STING-dependent recognition of cyclic di-AMP mediates type I interferon responses during Chlamydia trachomatis infection. MBio. (2013) 4:e00018–13. 10.1128/mBio.00018-13 23631912 PMC3663186

