# Supplementary material for: Selective Loss of Responsiveness to Exogenous but Not Endogenous Cyclic-Dinucleotides in Mice Expressing STING-R231H
Source: Front Immunol. 2020 Feb 21;11:238. doi: 10.3389/fimmu.2020.00238 (PMC7049784; doi:10.3389/fimmu.2020.00238)
Supplement: Supplementary file 1 [file Presentation_1.pptx]

## Slide 1
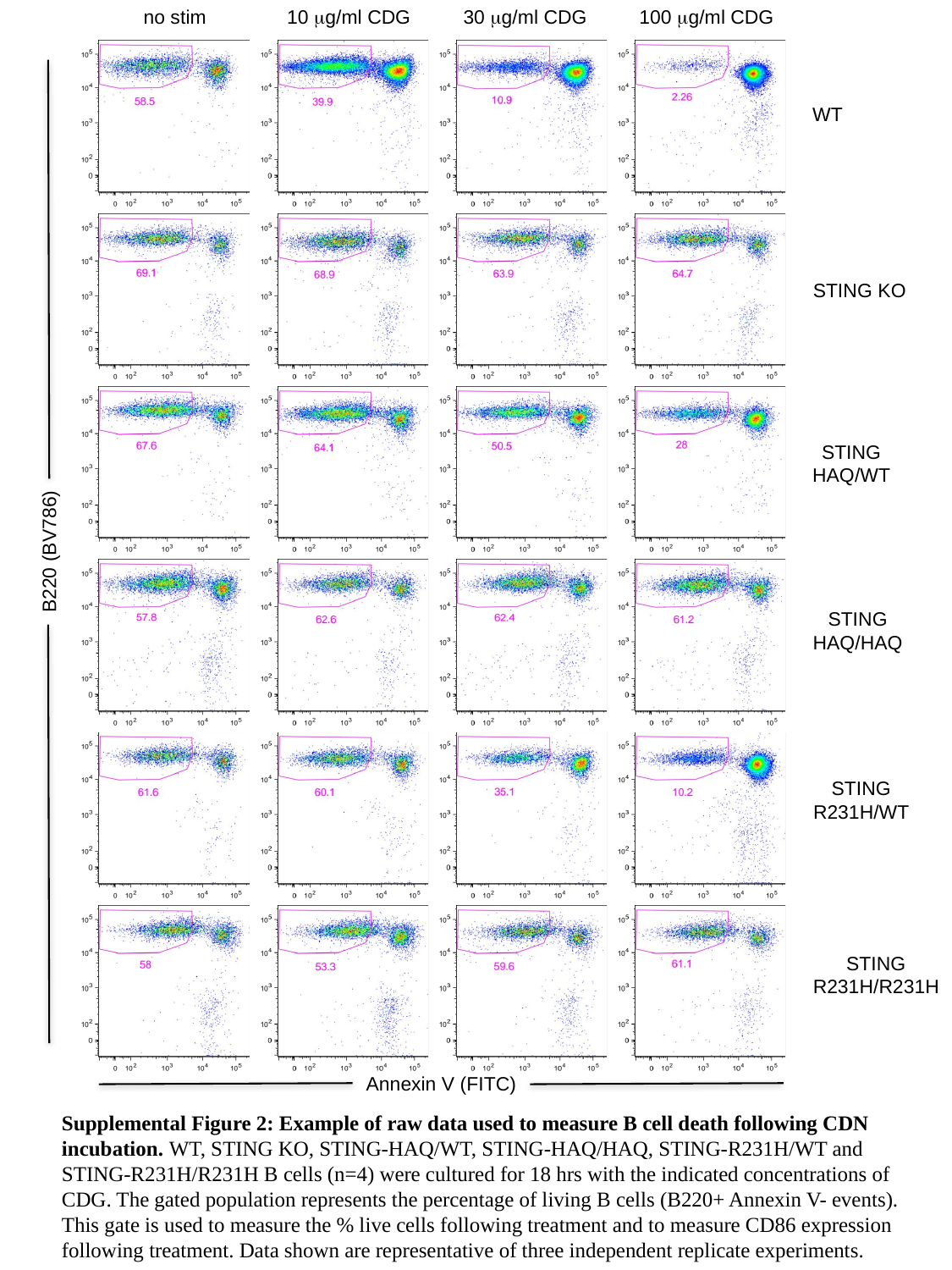

no stim
10 mg/ml CDG
30 mg/ml CDG
100 mg/ml CDG
WT
STING KO
STING
HAQ/WT
B220 (BV786)
STING
HAQ/HAQ
STING
R231H/WT
STING
R231H/R231H
Annexin V (FITC)
Supplemental Figure 2: Example of raw data used to measure B cell death following CDN incubation. WT, STING KO, STING-HAQ/WT, STING-HAQ/HAQ, STING-R231H/WT and STING-R231H/R231H B cells (n=4) were cultured for 18 hrs with the indicated concentrations of CDG. The gated population represents the percentage of living B cells (B220+ Annexin V- events). This gate is used to measure the % live cells following treatment and to measure CD86 expression following treatment. Data shown are representative of three independent replicate experiments.

## Slide 2
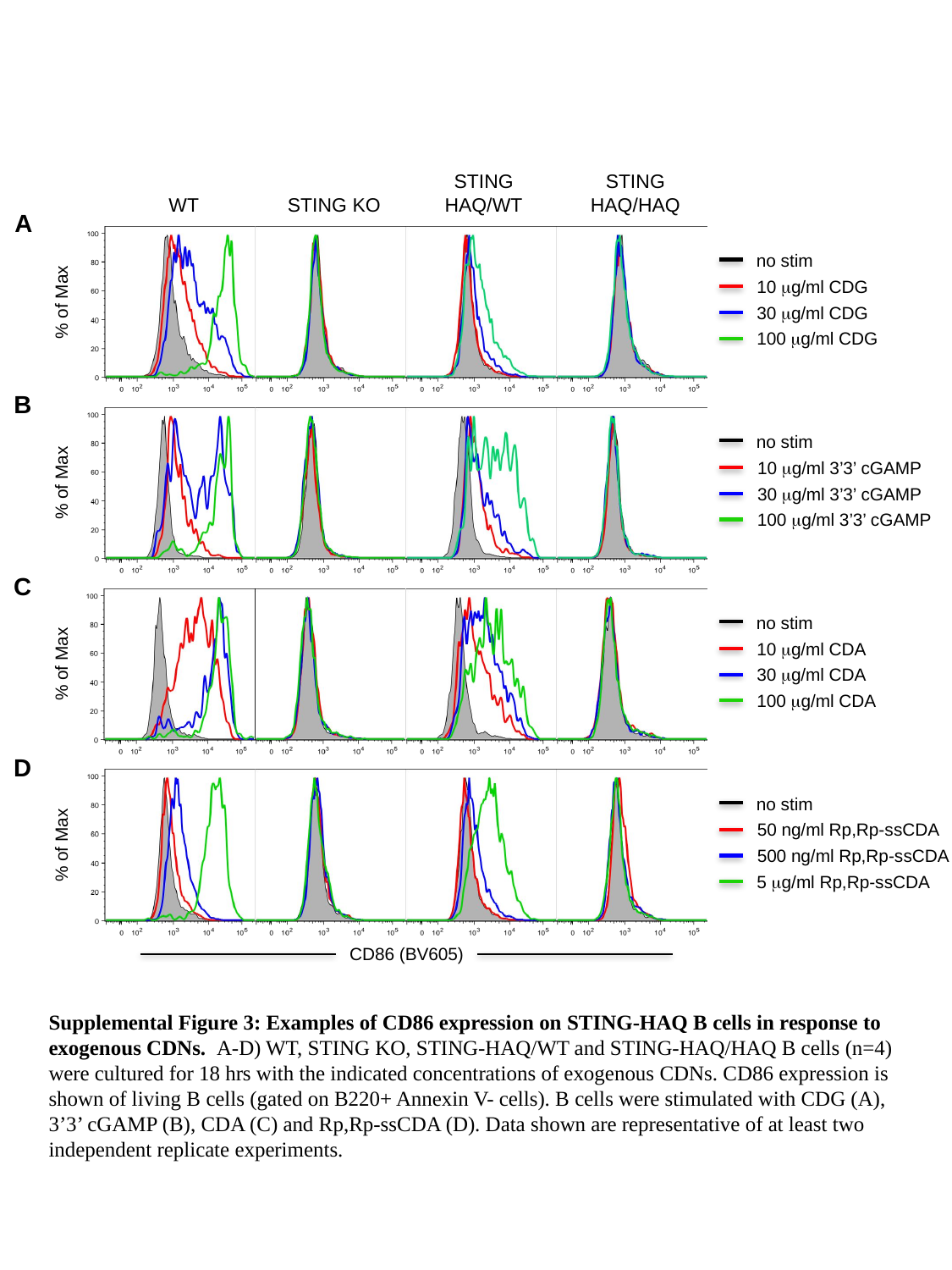

STING
HAQ/WT
STING
HAQ/HAQ
WT
STING KO
A
no stim
10 mg/ml CDG
% of Max
30 mg/ml CDG
100 mg/ml CDG
B
no stim
10 mg/ml 3’3’ cGAMP
% of Max
30 mg/ml 3’3’ cGAMP
100 mg/ml 3’3’ cGAMP
C
no stim
10 mg/ml CDA
% of Max
30 mg/ml CDA
100 mg/ml CDA
D
no stim
50 ng/ml Rp,Rp-ssCDA
% of Max
500 ng/ml Rp,Rp-ssCDA
5 mg/ml Rp,Rp-ssCDA
CD86 (BV605)
Supplemental Figure 3: Examples of CD86 expression on STING-HAQ B cells in response to exogenous CDNs. A-D) WT, STING KO, STING-HAQ/WT and STING-HAQ/HAQ B cells (n=4) were cultured for 18 hrs with the indicated concentrations of exogenous CDNs. CD86 expression is shown of living B cells (gated on B220+ Annexin V- cells). B cells were stimulated with CDG (A), 3’3’ cGAMP (B), CDA (C) and Rp,Rp-ssCDA (D). Data shown are representative of at least two independent replicate experiments.

## Slide 3
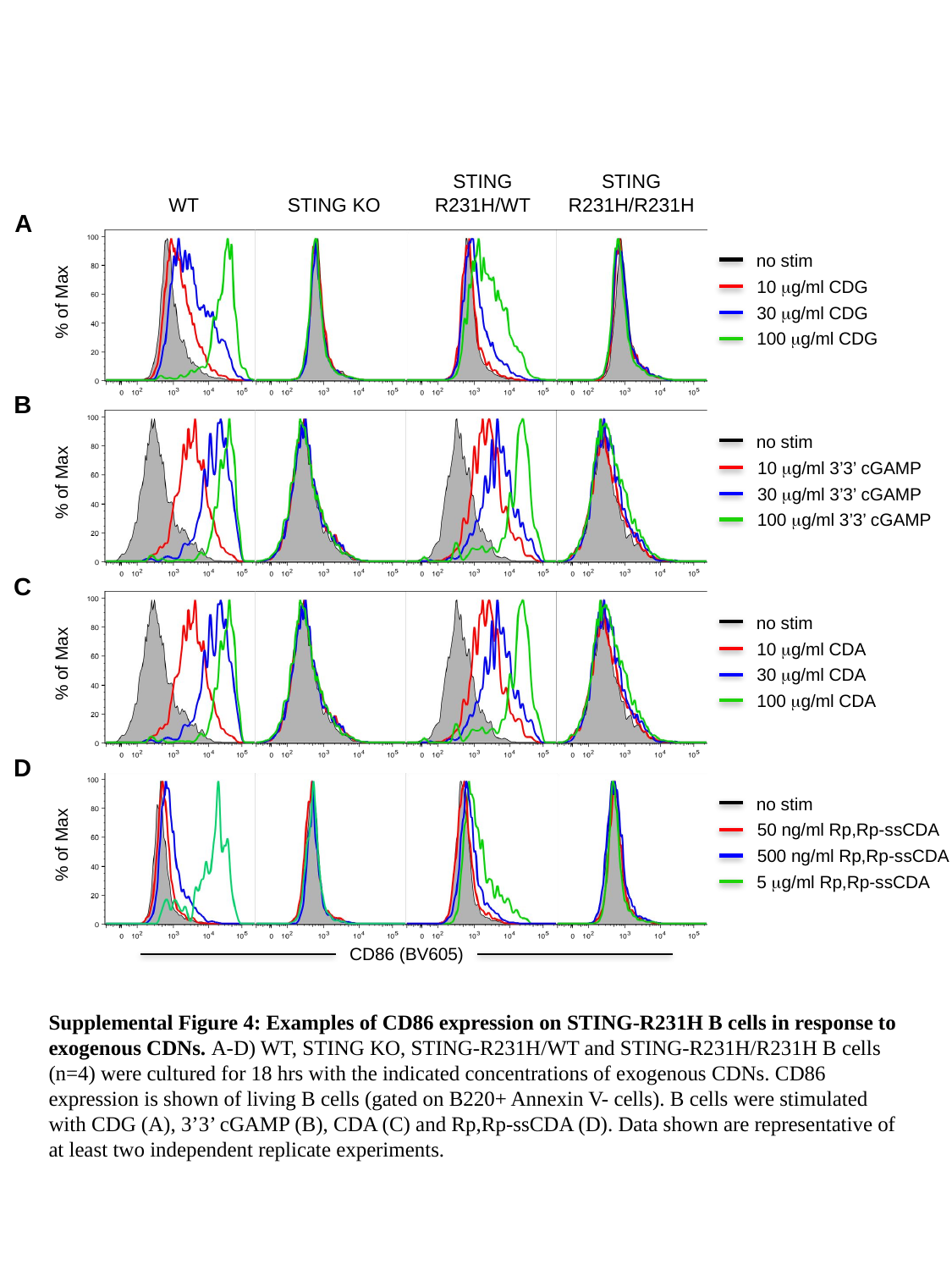

STING
R231H/WT
STING
R231H/R231H
WT
STING KO
A
no stim
10 mg/ml CDG
% of Max
30 mg/ml CDG
100 mg/ml CDG
B
no stim
10 mg/ml 3’3’ cGAMP
% of Max
30 mg/ml 3’3’ cGAMP
100 mg/ml 3’3’ cGAMP
C
no stim
10 mg/ml CDA
% of Max
30 mg/ml CDA
100 mg/ml CDA
D
no stim
50 ng/ml Rp,Rp-ssCDA
% of Max
500 ng/ml Rp,Rp-ssCDA
5 mg/ml Rp,Rp-ssCDA
CD86 (BV605)
Supplemental Figure 4: Examples of CD86 expression on STING-R231H B cells in response to exogenous CDNs. A-D) WT, STING KO, STING-R231H/WT and STING-R231H/R231H B cells (n=4) were cultured for 18 hrs with the indicated concentrations of exogenous CDNs. CD86 expression is shown of living B cells (gated on B220+ Annexin V- cells). B cells were stimulated with CDG (A), 3’3’ cGAMP (B), CDA (C) and Rp,Rp-ssCDA (D). Data shown are representative of at least two independent replicate experiments.

## Slide 4
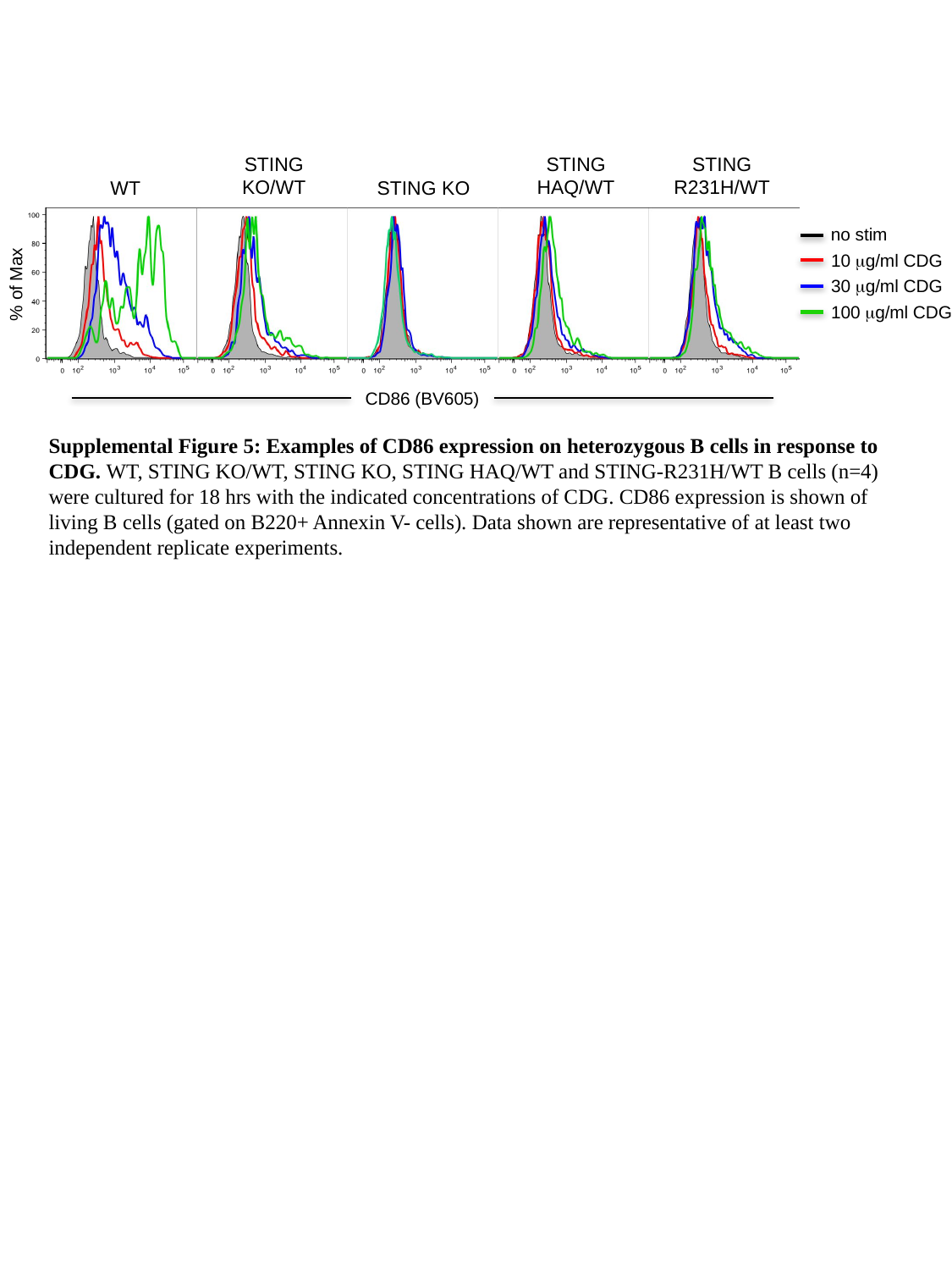

STING
KO/WT
STING
HAQ/WT
STING
R231H/WT
WT
STING KO
no stim
10 mg/ml CDG
% of Max
30 mg/ml CDG
100 mg/ml CDG
CD86 (BV605)
Supplemental Figure 5: Examples of CD86 expression on heterozygous B cells in response to CDG. WT, STING KO/WT, STING KO, STING HAQ/WT and STING-R231H/WT B cells (n=4) were cultured for 18 hrs with the indicated concentrations of CDG. CD86 expression is shown of living B cells (gated on B220+ Annexin V- cells). Data shown are representative of at least two independent replicate experiments.

## Slide 5
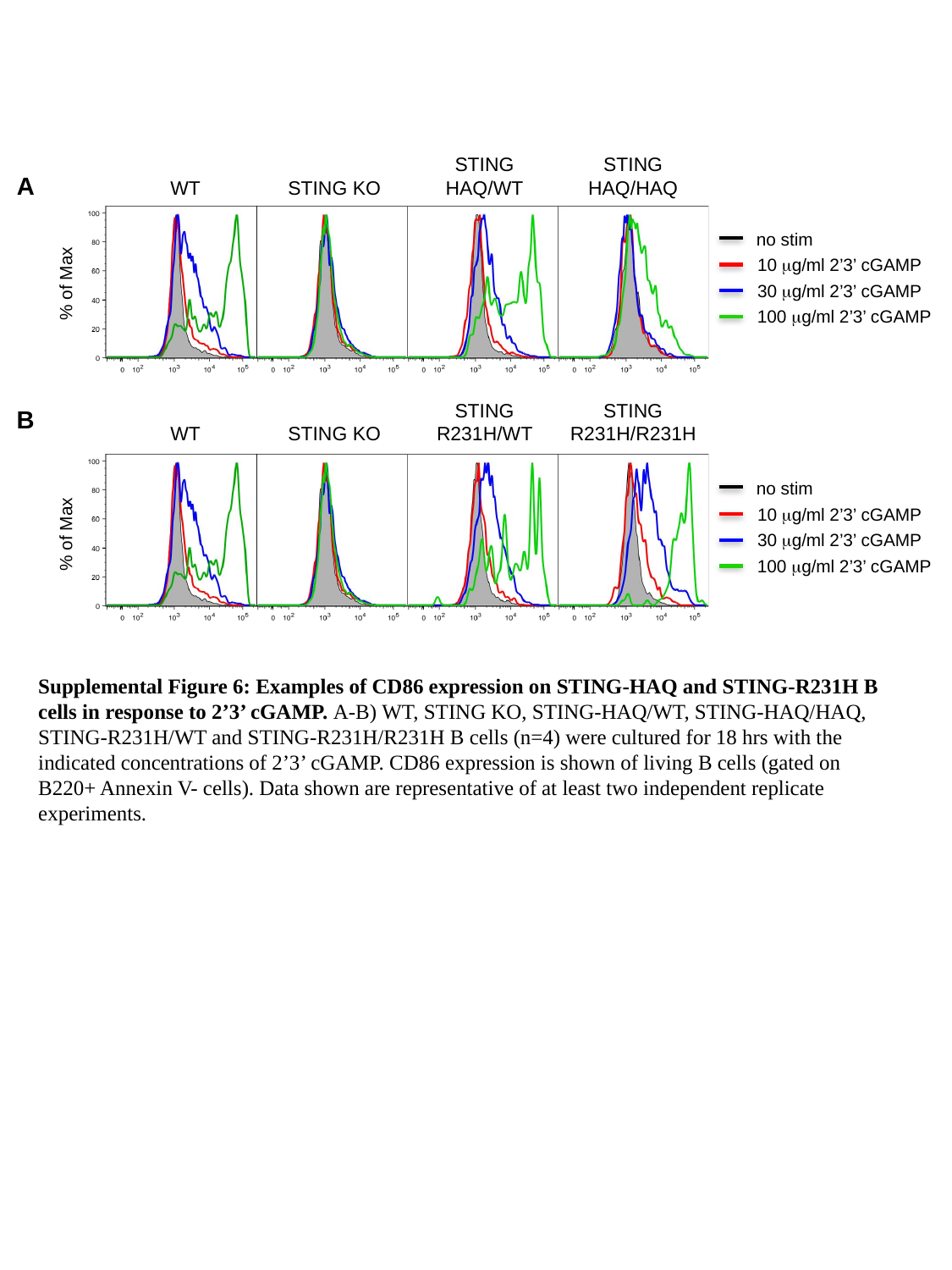

STING
HAQ/WT
STING
HAQ/HAQ
A
WT
STING KO
no stim
10 mg/ml 2’3’ cGAMP
30 mg/ml 2’3’ cGAMP
100 mg/ml 2’3’ cGAMP
% of Max
STING
R231H/WT
STING
R231H/R231H
B
WT
STING KO
no stim
10 mg/ml 2’3’ cGAMP
30 mg/ml 2’3’ cGAMP
100 mg/ml 2’3’ cGAMP
% of Max
Supplemental Figure 6: Examples of CD86 expression on STING-HAQ and STING-R231H B cells in response to 2’3’ cGAMP. A-B) WT, STING KO, STING-HAQ/WT, STING-HAQ/HAQ, STING-R231H/WT and STING-R231H/R231H B cells (n=4) were cultured for 18 hrs with the indicated concentrations of 2’3’ cGAMP. CD86 expression is shown of living B cells (gated on B220+ Annexin V- cells). Data shown are representative of at least two independent replicate experiments.
