# Supplementary material for: Selective Loss of Responsiveness to Exogenous but Not Endogenous Cyclic-Dinucleotides in Mice Expressing STING-R231H
Source: Front Immunol. 2020 Feb 21;11:238. doi: 10.3389/fimmu.2020.00238 (PMC7049784; doi:10.3389/fimmu.2020.00238)
Supplement: Supplementary file 2 [file Data_Sheet_1.PDF]

Supplementary Material

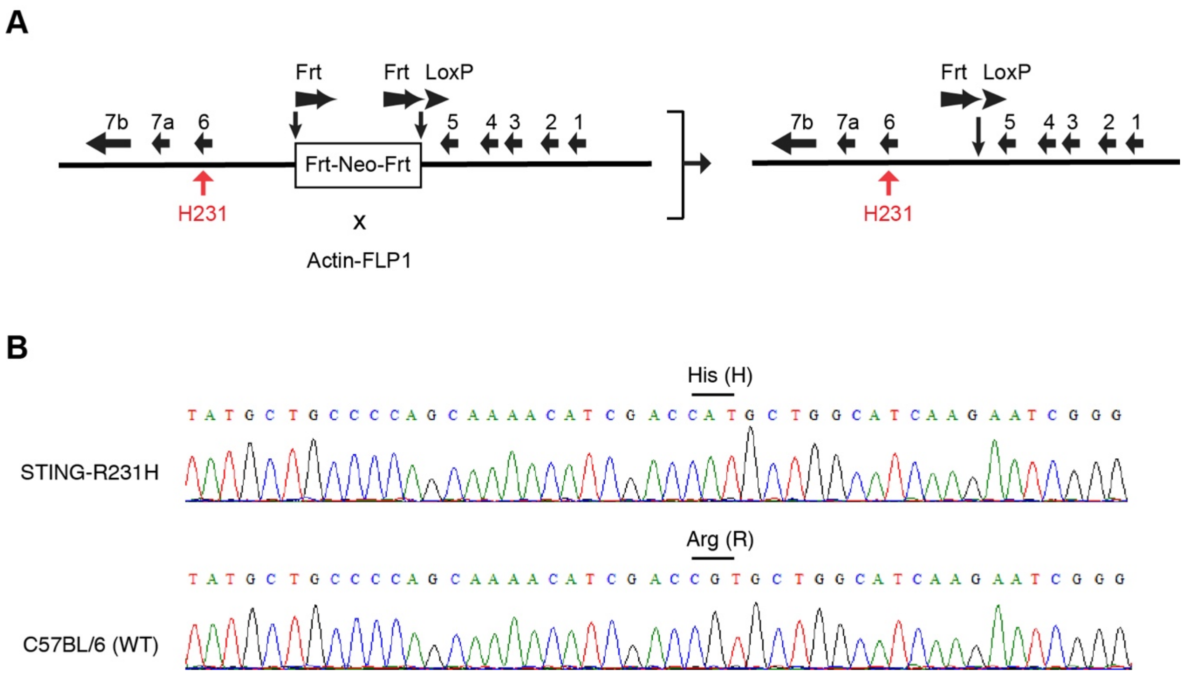

Supplementary Figure 1. Construction and validation of STING-R231H mice

A) Schematic representation of the STING locus following recombination with the targeting vector. The neo cassette was removed by crossing founder mice with a Flipase expressing strain. B) Sequencing of exon 6 from WT and STING-R231H/R231H mice to confirm the introduction of the G →A point mutation resulting in the R231H substitution.
